# Role of global aberrant alternative splicing events in papillary thyroid cancer prognosis

**DOI:** 10.18632/aging.101902

**Published:** 2019-04-15

**Authors:** Peng Lin, Rong-quan He, Zhi-guang Huang, Rui Zhang, Hua-yu Wu, Lin Shi, Xiao-jiao Li, Qing Li, Gang Chen, Hong Yang, Yun He

**Affiliations:** 1 Department of Medical Ultrasound, First Affiliated Hospital of Guangxi Medical University, Nanning, Guangxi Zhuang Autonomous Region 530021, P. R. China; 2 Department of Medical Oncology, First Affiliated Hospital of Guangxi Medical University, Nanning, Guangxi Zhuang Autonomous Region 530021, P. R. China; 3 Department of Pathology, First Affiliated Hospital of Guangxi Medical University, Nanning, Guangxi Zhuang Autonomous Region 530021, P. R. China; 4 Department of Cell Biology and Genetics, School of Pre-Clinical Medicine, Guangxi Medical University, Nanning, Guangxi 530021, P.R. China; 5 Departments of Pathology, Second Affiliated Hospital of Guangxi Medical University, Nanning, Guangxi 530021, P.R. China; 6 Departments of PET/CT, the First Affiliated Hospital of Guangxi Medical University, Nanning, P.R. China

**Keywords:** alternative splicing events, papillary thyroid cancer, precision medicine, prognostic signature, splicing factors

## Abstract

Background: Alternative splicing events have been increasingly reported for anomalous perturbations in various cancers, including papillary thyroid cancer (PTC).

Methods: Integration analysis of RNA sequencing and clinical information were utilized to identify survival associated splicing events in PTC. Then, several prognosis-related splicing events were submitted to develop moderate predictors for survival monitoring by using least absolute shrinkage and selection operator model. In addition, several biomedical computational algorithms were conducted to identify pathways enriched by genes with prognostic splicing events and construct regulatory network dominated by splicing factors.

Results: Survival analysis in 496 PTC patients indicated that TNM stage, tumor stage, distant metastasis and tumor status were significantly correlated with PTC patients' progression-free interval. 2799 splicing events were identified as prognostic molecular events. Functional enrichment analysis suggested that prognostic splicing events are associated with several energy metabolism-related processes. Based on these prognostic events, several prognostic signatures were developed. The final prognostic signature acted as an independent prognostic factor after adjusting for several clinical parameters. Interestingly, splicing regulatory network was constructed to display potential regulatory mechanisms of splicing events in PTC.

Conclusions: Our analysis provides the status of splicing events involved in the progression and may represent an underappreciated hallmark of PTC.

## Introduction

Recently, progress using high-throughput sequencing technologies has spurred cancer genome research. Strikingly, the limited number of protein-coding genes makes it difficult to account for the abundant proteomic phenotypes, especially for tumors [1,2]. Alternative splicing (AS) is a major physiological phenomenon that allows for transcript variants in mammalian cells and for subsequent reprogramming of protein diversity for better environmental fit [3]. Given the indispensable functional impact of AS in genomic analysis, it is not surprising that disruptions of AS often lead to aberrant cellular homeostasis and are linked to cancer. Characterization of the AS landscape in cancers via reliable big data to identify biomarkers provides a wealth of insight into cancers with excellent prognostic value. Hence, increasing evidence has indicated that AS is actively involved in the initiation, progression and prognosis of cancers [4,5]. Additionally, cancer researchers realize the significant clinical utility of AS and its potential as a useful therapeutic signaling target [6–8]. In general, AS is a complex and tightly regulated process orchestrated by limited splicing factors (SFs) [9]. Therefore, recent analyses of cancer genomes have predominantly focused on evaluating the clinical significance of AS events and SFs in tumors, their potentially pathogenic impact for downstream pathways and the development of their regulatory network.

Thyroid cancer is the most common endocrine neoplasia, where papillary thyroid cancer (PTC) represents more than 90% of all thyroid cancers [10]. In recent decades, the sharp increase in PTC morbidity has aroused substantial concern [11,12]. Although PTC is indolent in most cases, high-incidence patients develop distant metastasis and lymph node metastasis, which often lead to high mortality. Therefore, it is imperative to screen for biomarkers to characterize PTC recurrence and metastasis for effective prognostic monitoring. AS is exploited by tumor cells, allowing the cells to live by sustaining a series of malignant behaviors. AS is emerging as an important diagnostic and prognostic signature to further understand tumorigenesis and to develop new targets for precision therapy in PTC [13]. To elucidate the global set of AS events, their contribution to the onset and development of PTC and how they affect molecular signaling to exert their biological function, a comprehensive analysis based on large clinical samples is urgently needed. RNA sequencing (RNA-seq) data generated by The Cancer Genome Atlas (TCGA) have supported data at the genome-wide level to identify the prognostic value of AS. Cancer-specific AS events could be effective biomarkers for clinical monitoring; however, dysregulation of splicing patterns on a transcriptome-wide scale has been less well studied until recently in PTC.

To unravel the pattern of global aberrant AS and its clinical implications in PTC, we focused on global AS patterns with complete clinical information from the TCGA database. From the perspective of AS, prognostic risk score systems were constructed based on AS events to predict the prognosis of PTC. Furthermore, we study how biological processes are affected by these AS evens. To assess the regulatory relationships between AS events and SFs, an evaluation of the regulatory network in silico tools is also presented.

## RESULTS

### Cohort characteristics

We processed TCGA splice-seq files and clinical information of PTC patients. A total of 496 patients were included in the present analysis. The general clinical characteristics are summarized in Table 1. Before exploring the prognostic values of AS events, univariate Cox hazard analyses were performed to assess the relationship between the clinical features and clinical outcome of PTC patients (Table 1). American Joint Committee on Cancer (AJCC) TNM stage (hazard ration (HR)=2.780, 95% CI: 1.582-4.886; P<0.001), tumor stage (HR=2.830, 95% CI: 1.583-5.060; P<0.001), distant metastasis (HR=6.432, 95% CI: 2.202-18.789; P =0.001) and tumor status (HR=16.566, 95% CI: 9.361-29.314; P<0.001) were significantly correlated with the progression-free interval (PFI) of PTC. However, no significant correlations were observed between PFI and age, gender, lymph node metastasis and neoplasm focus type.

**Table 1:** General characteristics of included papillary thyroid cancer patients.

Clinical parameters		No.	PFI		Hazard rations (95%CI)	P value
			Event	Censored		
All		483	49	434	-	-
Age	≥50	214	27	187	1.705 (0.969-2.998)	0.064
	<50	269	22	247		
Gender	Male	128	18	110	1.757 (0.982-3.142)	0.058
	Female	355	31	324		
AJCC TNM stage	Stage III/IV	158	27	131	2.780 (1.582-4.886)	<0.001
	Stage I/II	324	22	302		
	NA	1	0	1		
Tumor stage	T3-T4	184	31	153	2.830 (1.583-5.060)	<0.001
	T1-T2	297	18	279		
	NA	2	0	2		
Lymph node metastasis	Positive	212	28	184	1.713 (0.938-3.131)	0.080
	Negative	222	17	205		
	NA	49	4	45		
Distant metastasis	Positive	8	4	4	6.432 (2.202-18.789)	0.001
	Negative	273	22	251		
	NA	202	23	179		
Neoplasm focus type	Multifocal	214	21	193	1.101 (0.623-1.947)	0.740
	Unifocal	259	28	231		
	NA	10	0	10		
Tumor status	With tumor	40	25	15	16.566 (9.361-29.314)	<0.001
	Tumor free	434	23	411		
	NA	9	1	8		

PFI: progression-free interval; NA: not available; CI: confidence interval.

### Survival associated AS events

**Figure 1:**
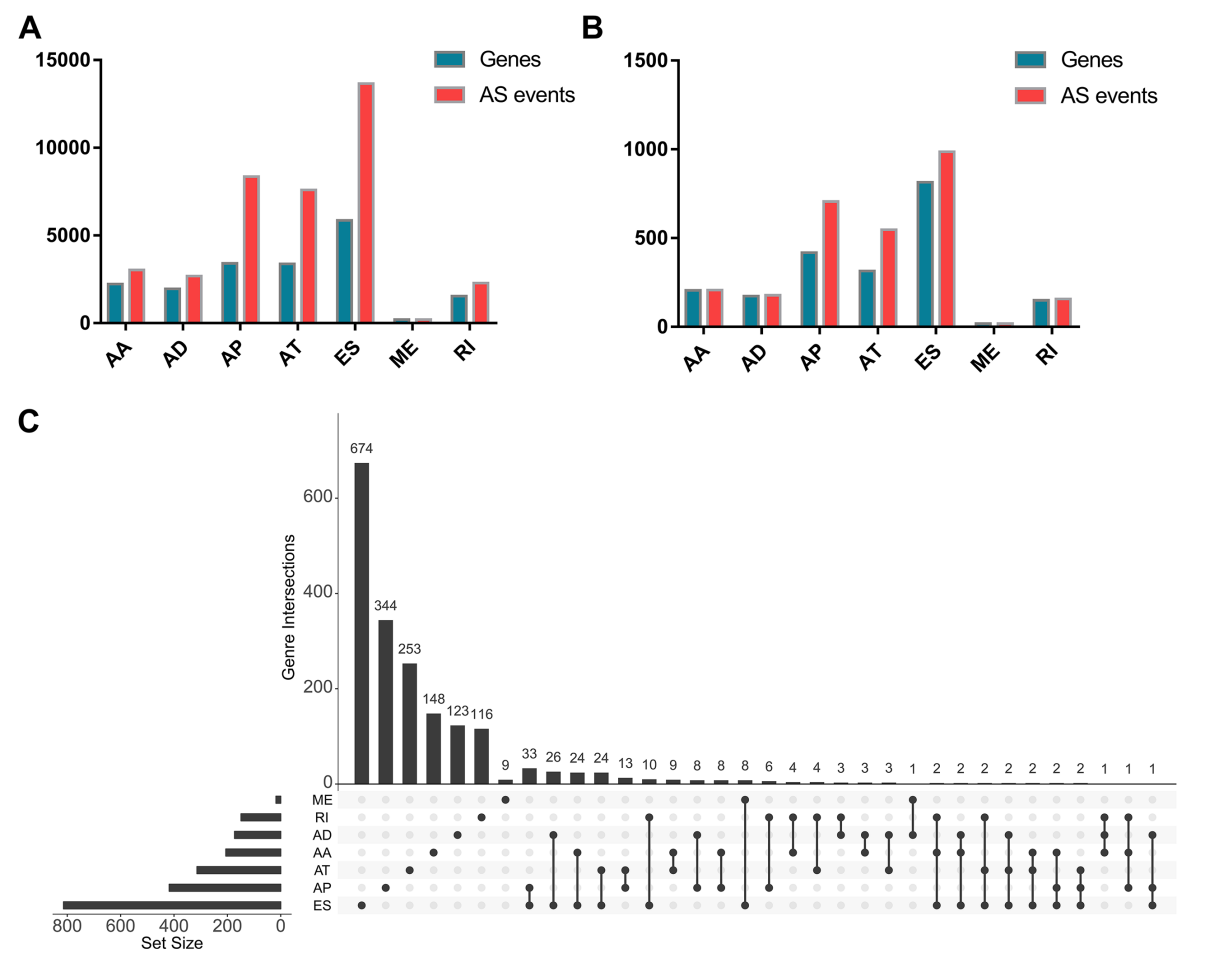
**Prognosis-related alternative splicing (AS) events.** (**A**) The number of AS events and corresponding genes included in the present study; (**B**) The number of prognosis-related AS events and corresponding genes obtained by using univariate COX analysis; (**C**) UpSet plots in papillary thyroid cancer, showing the interactions among the seven types of AS events. One gene may have up to three types of AS events.

As a whole, there are 3025 alternate acceptor (AA) events in 2227 genes, 2683 alternate donor (AD) events in 1955 genes, 8351 alternate promoter (AP) events in 3409 genes, 7598 alternate terminator (AT) events in 3381 genes, 13655 exon skip (ES) events in 5855 genes, 209 mutually exclusive exon (ME) events in 202 genes, and 2280 retained intron (RI) events in 1548 genes for evaluation of prognostic value (Figure 1A). A total of 207 AA events in 206 genes, 178 AD events in 173 genes, 706 AP events in 418 genes, 547 AT events in 314 genes, 986 ES events in 814 genes, 18 ME events in 18 genes, and 157 RI events in 149 genes were identified as prognosis-associated AS events (P<0.05) (Figure 1B). Thus, one gene might have two or more AS events that were markedly related to the PFI of PTC patients. The UpSet plot vividly revealed that ES was the most common prognosis-related event, and a gene could have up to three prognosis-related events (Figure 1C).

### Molecular characteristics of survival-associated AS events

**Figure 2:**
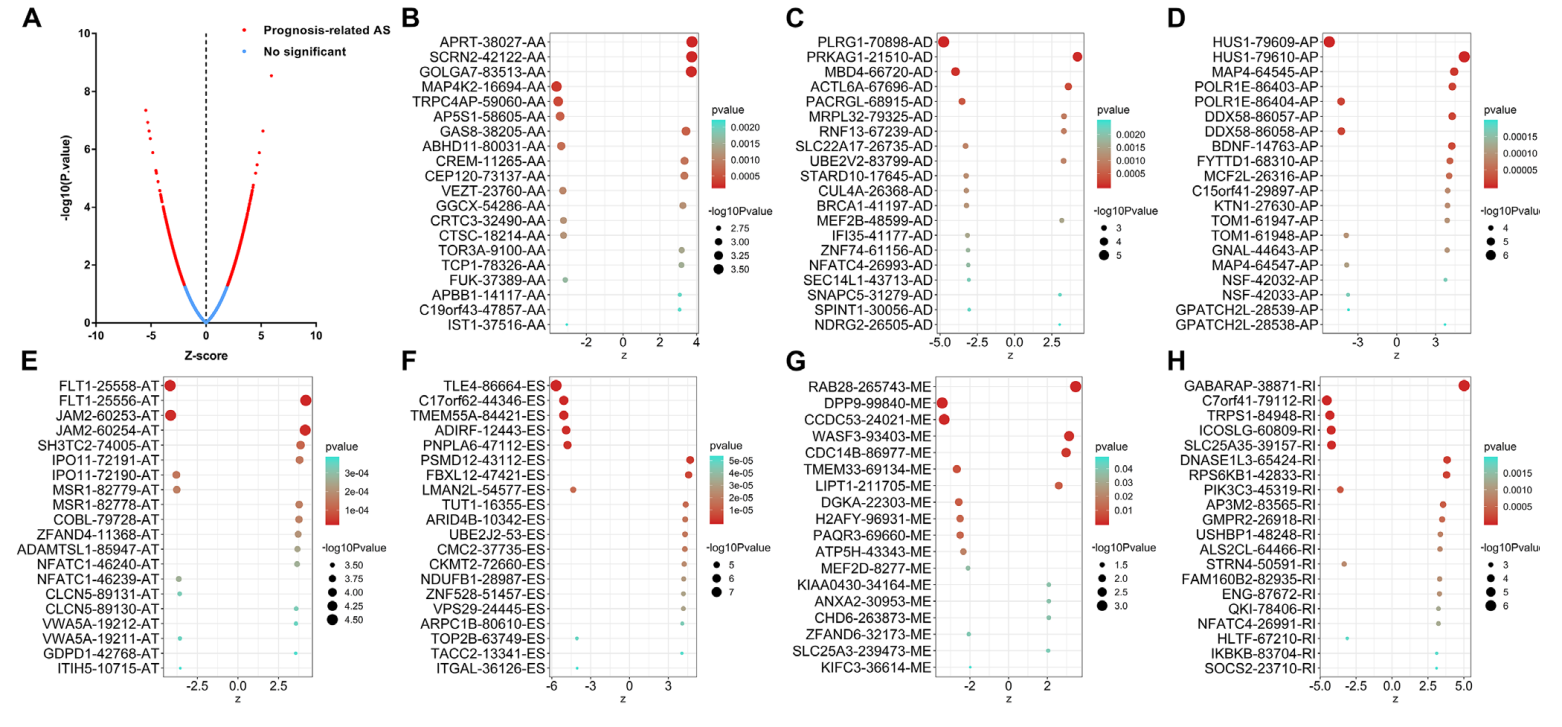
**Top 20 most significant alternative splicing (AS) events in papillary thyroid cancer.** (**A**) The red dots represent AS events that are significantly correlated with patient survival. The blue dots represent AS events without correlation. The top 20 AS events correlated with clinical outcome based on acceptor sites (**B**), alternate donor sites (**C**), alternate promoters (**D**), alternate terminators (**E**), exon skips (**F**), mutually exclusive exons (**G**), and retained introns (**H**).

**Figure 3:**
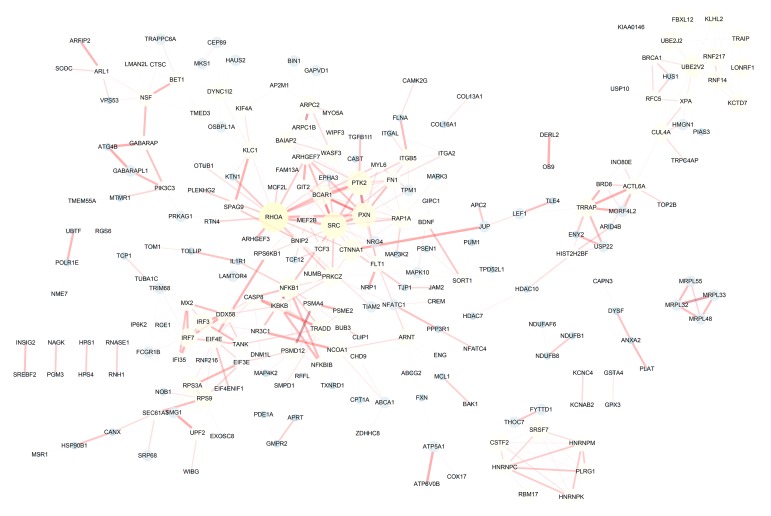
**Protein-protein interaction network of genes with survival-associated alternative splicing events in papillary thyroid cancer.** The bigger the point in the network, the more important it is.

**Figure 4:**
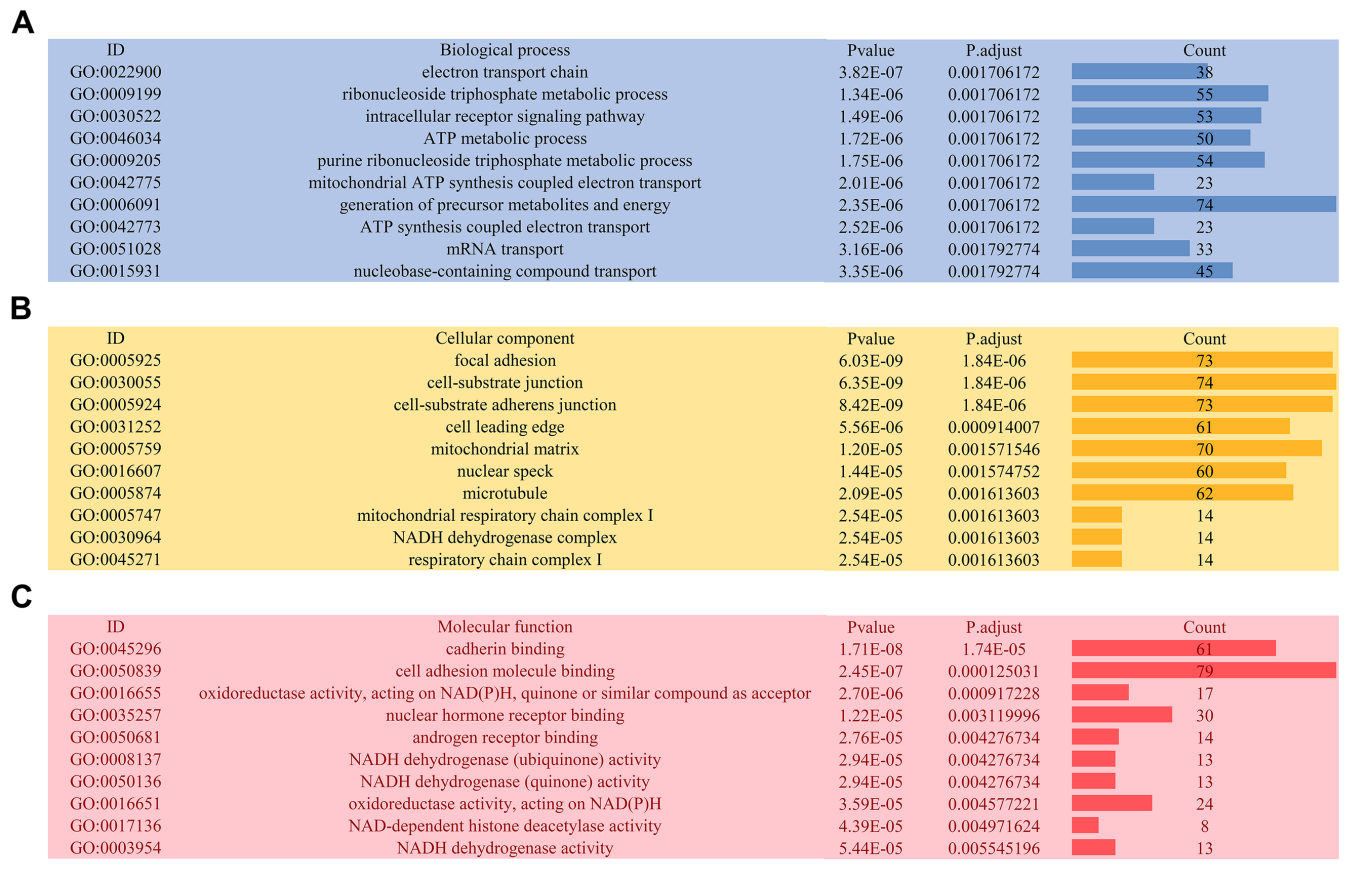
**Gene ontology analysis of genes with survival-associated alternative splicing events.** (**A**) Biological process; (**B**) Cellular component; (**C**) Molecular function.

**Figure 5:**
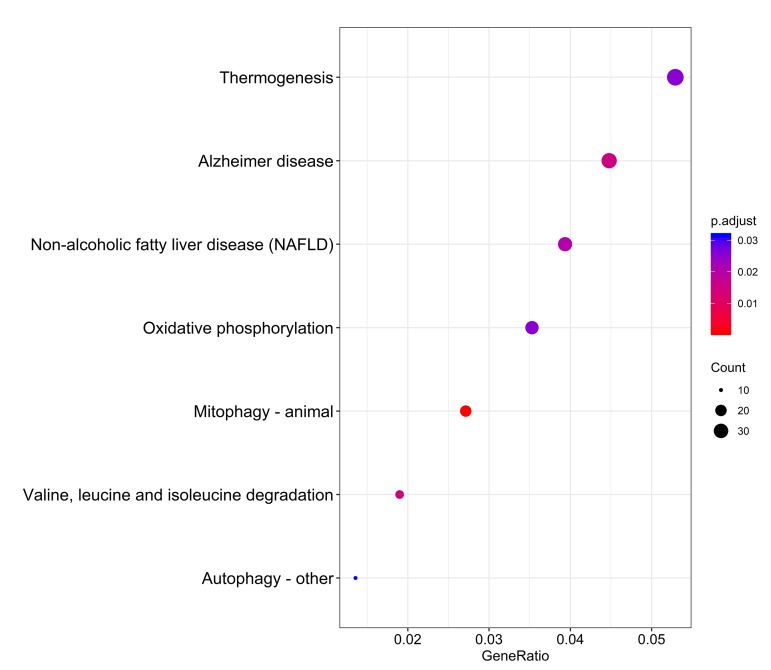
Kyoto Encyclopedia of Genes and Genomes (KEGG) pathway analysis of genes with survival-associated alternative splicing events.

The distributions of AS events significantly correlated with patient survival are displayed in Figure 2A. The 20 most significant prognosis-related AS events are also shown (Figure 2B-H). Among them, there were only 18 prognostic M events. To reveal the molecular characteristics of genes with survival-associated AS events, several bioinformatics analyses were conducted. First, a PPI network was constructed to demonstrate the relationships among these genes. RHOA, SRC, PXN and PTK2 ranked at the core in the network (Figure 3). According to the functional annotations of clusterProfiler, “electron transport chain”, “ribonucleoside triphosphate metabolic process” and “intracellular receptor signaling pathway” were the three most significant biological process terms (Figure 4A). “Focal adhesion”, “cell-substrate junction” and “cell-substrate adherens junction” were the three most significant cellular component terms (Figure 4B). For molecular function, “cadherin binding”, “cell adhesion molecule binding” and “oxidoreductase activity, acting on NAD(P)H, quinone or similar compound as acceptor” were three most enriched categories (Figure 4C). More importantly, we found that the “thermogenesis” pathway was correlated with these genes most significantly (Figure 5).

### Prognostic signatures for PTC patients

**Figure 6:**
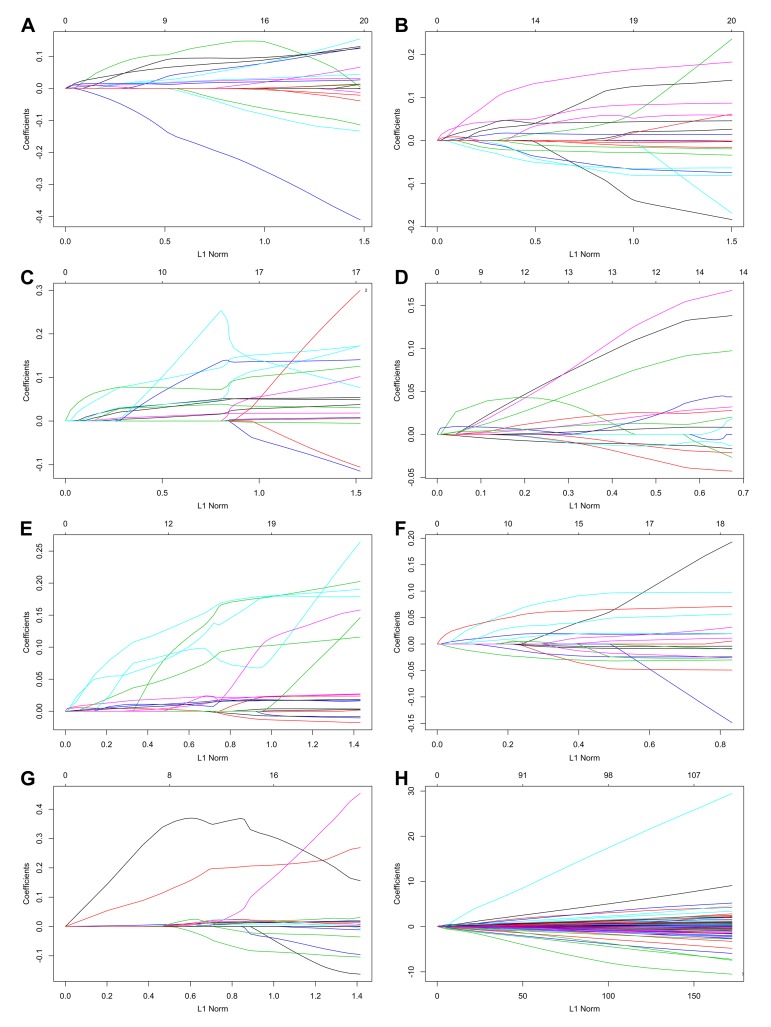
Construction of prognostic signatures based on LASSO COX analysis.

**Figure 7:**
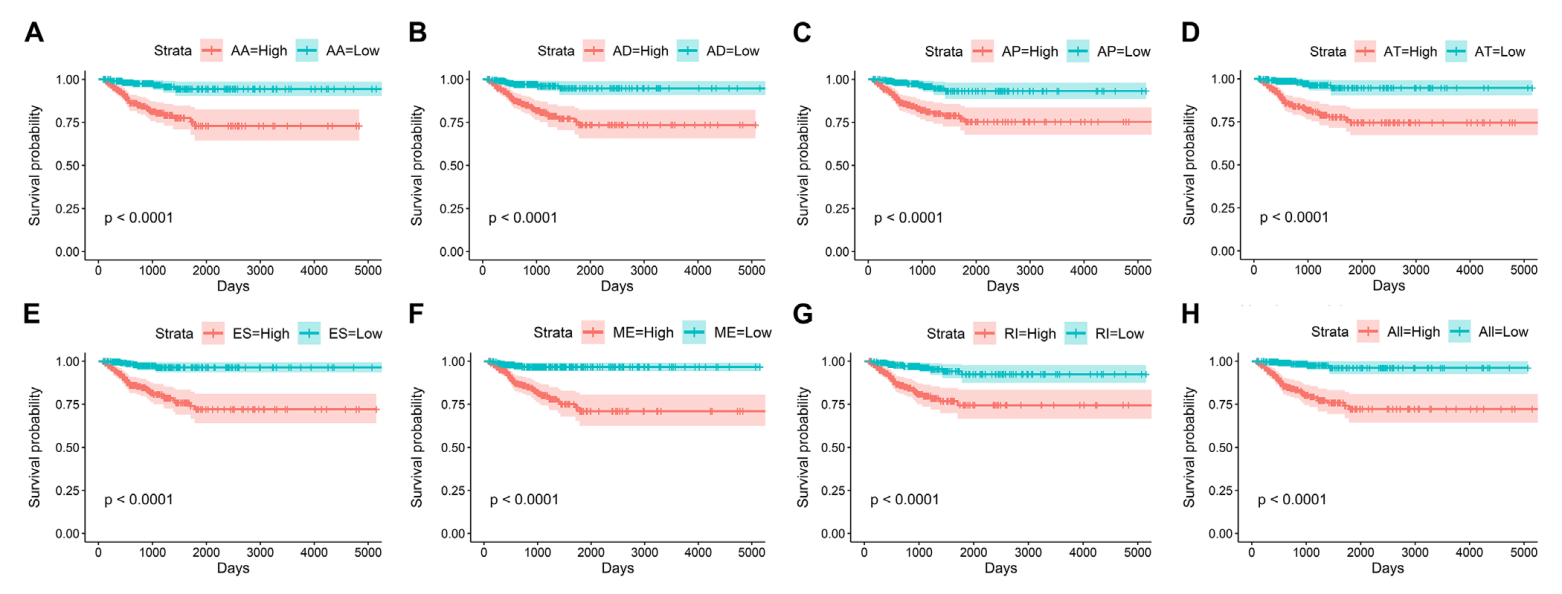
Kaplan-Meier curves of prognostic predictors for papillary thyroid cancer.

**Figure 8:**
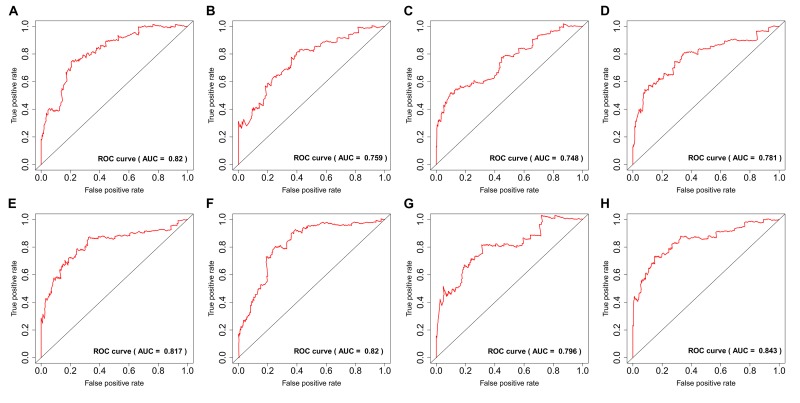
ROC curves of prognostic predictors for papillary thyroid cancer.

**Figure 9:**
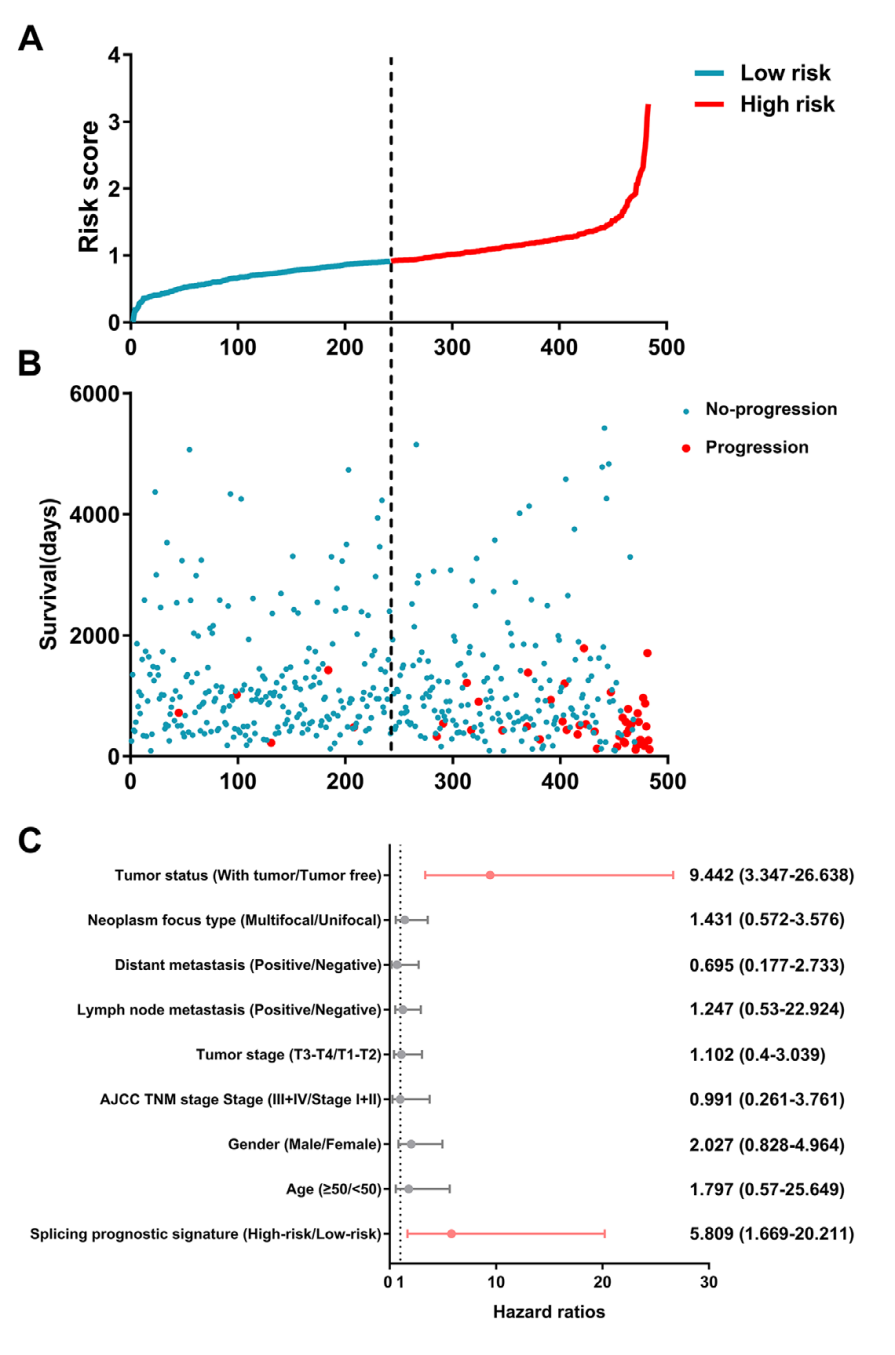
Identification capability of prognostic signature for separating patients into high- and low-risk groups.

By using the least absolute shrinkage and selection operator (LASSO) Cox analysis following univariate Cox, we developed seven types of prognostic signatures based on AA, AD, AP, AT, ES, ME and RI (Figure 6, Table 2). Furthermore, we selected the top 20 most significant survival-associated AS events in the seven types to construct the final prognostic signature (Table 3). Interestingly, we found that the seven prognostic signatures could predict the clinical outcome of PTC patients (Figure 7). ROC curves validated the performance of prognostic signatures in prognosis prediction (Figure 8). The final prognostic signature was the most ideal predictor (Figure 9A). This signature could distinguish the PTC patients with distinct clinical outcome very well (Figure 9B). After multivariate adjustment by clinical factors, the prognostic signature remained a moderate and independent prognostic indicator (HR=5.809, 95%CI:1.669-20.211, P<0.001; Figure 9C).

**Table 2:** Prognostic signatures for papillary thyroid cancer.

Type	Formula	Hazard ratio (95%CI)	AUC
AA	APBB1-14117-AA * 0.104543 + APRT-38027-AA * 0.03133 + C19orf43-47857-AA * 0.025953 + CREM-11265-AA * 0.086061 + FUK-37389-AA * -0.123280 + GAS8-38205-AA * 0.01930 + GGCX-54286-AA * 0.016141 + GOLGA7-83513-AA* 0.063632 + SCRN2-42122-AA * 0.013288	5.767 (3.291-10.110)	0.820
AD	IFI35-41177-AD * (-0.000762) + MBD4-66720-AD * (-0.018721) + MEF2B-48599-AD * 0.062115 + MRPL32-79325-AD * 0.01512 + NDRG2-26505-AD * 0.002702 + PRKAG1-21510-AD * 0.040404 + RNF13-67239-AD * 0.026105 + SLC22A17-26735-AD * (-0.01058) + SNAPC5-31279-AD * 0.009076	5.526 (3.155-9.678)	0.759
AP	BDNF-14763-AP * 0.019345 + DDX58-86057-AP * 0.010935 + FYTTD1-68310-AP * 0.054980 + GNAL-44643-AP * 0.00322 + GPATCH2L-28538-AP * 0.011095 + HUS1-79610-AP * 0.069287 + MAP4-64545-AP *0.019803 + MCF2L-26316-AP * 0.000561	4.803 (2.742-8.412)	0.748
AT	ADAMTSL1-85947-AT * 0.103877 + CLCN5-89130-AT * 0.024612 + COBL-79728-AT * 0.005752 + GDPD1-42768-AT * 0.118004 + IPO11-72190-AT * (-0.01062) + ITIH5-10715-AT * (-0.010241) + JAM2-60254-AT * 0.012414 + MSR1-82778-AT * 0.011844 + MSR1-82779-AT * (-0.013158) + NFATC1-46240-AT * 0.018544 + SH3TC2-74005-AT * 0.005534 + VWA5A-19211-AT * (-0.021799) +ZFAND4-11368-AT * 0.070475	6.256 (3.573-10.950)	0.781
ES	ARID4B-10342-ES * 0.003164 + ARPC1B-80610-ES * 0.079956 + CKMT2-72660-ES * 0.010942 + CMC2-37735-ES * 0.125418 + FBXL12-47421-ES * 0.01949 + NDUFB1-28987-ES * 0.04923 + PSMD12-43112-ES * 0.079472 + TACC2-13341-ES * 0.00426 + TUT1-16355-ES * 0.010645 + UBE2J2-53-ES * 0.086546 + VPS29-24445-ES * 0.000217 + ZNF528-51457-ES * 0.008070	7.805 (4.456-13.670)	0.817
ME	CCDC53-24021-ME * (-0.017563) + CDC14B-86977-ME * 0.012660 + CHD6-263873-ME * 0.019448 + KIFC3-36614-ME * (-0.003343) + LIPT1-211705-ME * 0.043762 + RAB28-265743-ME * 0.042571 + WASF3-93403-ME * 0.006051	6.750 (3.852-11.830)	0.820
RI	ALS2CL-64466-RI * 0.003951 + AP3M2-83565-RI * 0.189298 + C7orf41-79112-RI * -0.036867 + DNASE1L3-65424-RI * 0.011210 + GABARAP-38871-RI * 0.355941 + GMPR2-26918-RI * 0.018861 + IKBKB-83704-RI * 0.016560 + NFATC4-26991-RI * 0.015686 + RPS6KB1-42833-RI * 0.01487 + SOCS2-23710-RI * 0.003253 + USHBP1-48248-RI * 0.011626	4.794 (2.730-8.418)	0.796
All	BDNF-14763-AP * 0.016836 + DDX58-86057-AP * 0.010816 + FYTTD1-68310-AP * 0.050450 + GNAL-44643-AP * 0.002647 + GPATCH2L-28538-AP * 0.008745 + HUS1-79610-AP * 0.04913 + MAP4-64545-AP * 0.017245 + IPO11-72190-AT * -0.001612 + ZFAND4-11368-AT * 0.006298 + CKMT2-72660-ES * 0.004576 + CMC2-37735-ES * 0.069065 + FBXL12-47421-ES * 0.007271 + NDUFB1-28987-ES * 0.019385 + PSMD12-43112-ES * 0.012527 + ZNF528-51457-ES * 0.002064 + AP3M2-83565-RI * 0.015821 + DNASE1L3-65424-RI * 0.001367 + GABARAP-38871-RI * 0.044678	9.841 (5.616-17.240)	0.843

**Table 3:** Prognostic predictors for papillary thyroid cancer patients.

Gene	As id	Splice type	Exons	Univariate COX		Index
				HR	P-value	
BDNF	14763	AP	1.2:1.3:1.4:1.5	1.048	2.08E-05	0.016836
DDX58	86057	AP	1	1.092	1.87E-05	0.010816
FYTTD1	68310	AP	2	1.103	4.07E-05	0.050450
GNAL	44643	AP	4.2:4.3:4.4	1.021	9.88E-05	0.002647
GPATCH2L	28538	AP	2	1.061	0.000195096	0.008745
HUS1	79610	AP	9.2	1.150	1.86E-07	0.049129
MAP4	64545	AP	3.1:3.2	1.053	9.30E-06	0.017245
IPO11	72190	AT	1	0.932	0.000146324	-0.001612
ZFAND4	11368	AT	1.2:1.3:1.4	1.124	0.000205212	0.006298
CKMT2	72660	ES	1	1.025	2.37E-05	0.004576
CMC2	37735	ES	3.1	1.189	1.90E-05	0.069065
FBXL12	47421	ES	2.1	1.050	4.93E-06	0.007271
NDUFB1	28987	ES	32	1.108	2.81E-05	0.019385
PSMD12	43112	ES	13	1.203	2.84E-06	0.012527
ZNF528	51457	ES	3	1.037	3.10E-05	0.002064
AP3M2	83565	RI	2:03	1.280	0.00037889	0.015821
DNASE1L3	65424	RI	6	1.023	0.000124882	0.001368
GABARAP	38871	RI	9	2.469	5.19E-07	0.044678

### Survival-associated SF-AS network

**Figure 10:**
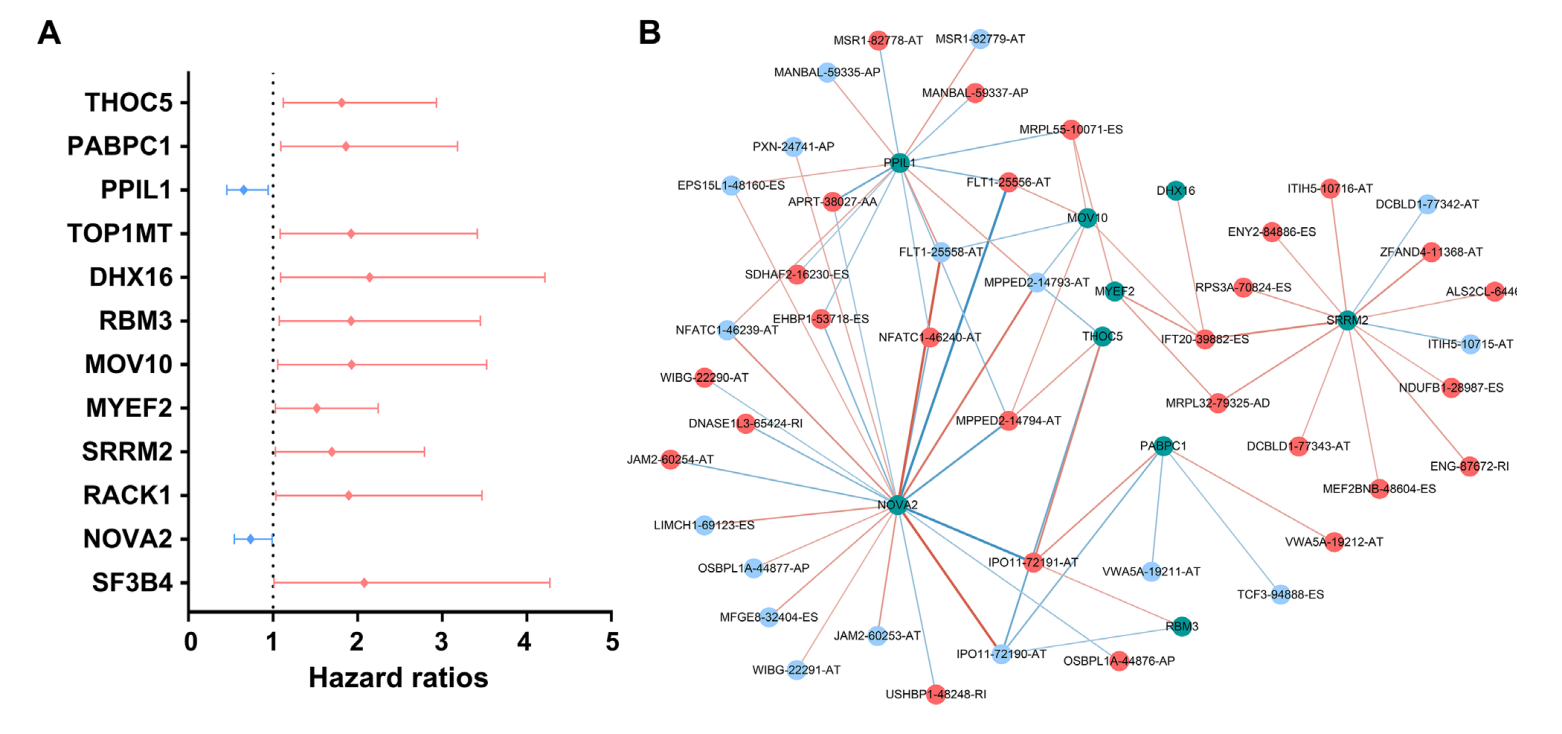
**Survival-associated splicing factors and splicing correlation network in papillary thyroid cancer.** (**A**) Survival-associated alternative splicing events; (**B**) Alternative splicing events whose PSI values were positively/negatively correlated with survival times are represented with red/blue dots. Green dots are survival-associated splicing factors. The positive/negative correlations between expressions of splicing factors and PSI values for alternative splicing are represented with red/blue lines.

AS events are mainly orchestrated by SFs, which often bind to pre-mRNAs and regulate RNA splicing via influencing exon selection and splicing site. Therefore, exploration of the SF-AS regulatory network is imperative in PTC. Survival analyses based on TCGA data suggested that 12 SFs possess the ability to predict the PFI of PTC patients (Figure 10A). Next, correlation analyses between the expression of SFs and PSI value of the most significant AS events (P<0.0001) were conducted (Figure 10B).

## DISCUSSION

Currently, scientific research on the role of AS events in PTC still has many unanswered questions owing to the dearth of available large-sample public AS profiles and the paucity of systematic analysis referring to their clinical significance and deep molecular function. These bottlenecks have prevented cancer researchers from effectively recognizing the widespread applicability of AS events in PTC. Exploration of AS patterns broadens our vision and our understanding in traditional transcriptome molecular biomarkers. In this project, we adopted several biomedical computational approaches, which integrate the AS event profiles and clinical information of PTC patients to mine prognosis-related AS and construct splicing prognostic signatures that could stratify PTC patients into subgroups with distinct survival outcomes. Moreover, the SF-AS network could provide further insights into regulatory mechanisms in patients with PTC from the perspective of splicing.

PTC are characterized as an indolent disease, effective biomarkers that predictive the clinical outcome is limited. And the effectiveness prophylactic central compartment lymph node dissection in PTC is still controversial. The prophylactic dissection should be avoided to reduce surgical complications after thyroidectomy [14,15]. Hence, it is imperative to precise prediction instead of potential “overtreatment”. In recent years, next-generation sequencing technology has extensively promoted the investigation of whole-genome analyses, including genome splicing exploration. Previously, several studies conducted SpliceSeq analyses to generate alterative splicing profiles for some types of cancer, as well as to construct prognostic signatures for cancer prognosis monitoring, including non-small cell lung cancer [16], ovarian cancer [17], bladder cancer [18], and gastrointestinal cancer [19]. To the best of our knowledge, we are the first group to integrate splicing profiles and TCGA clinical factors of PTC patients together to comprehensively investigate the prognostic value of AS. This computational bioinformatics pipeline could provide novel insights into the clinical value and potential regulatory mechanisms of AS at the genome-wide level. Previously, several studies have proposed transcriptomic signatures associated with the carcinogenesis and aggressiveness of PTC [20,21]. The present in-depth study further explored alterations of transcriptomes used as prognostic predictors and could broaden our horizons in the clinical significance of transcriptomic signatures.

Given the multitude of AS events impacted by their own pre-mRNAs, the downstream functional impact is partly used to describe the molecular function of AS alteration events. In the PPI network analysis, RHOA, SRC, PXN and PTK2 were the hub genes. Notably, RHOA and SRC have both been identified as important molecules involved in the biological process of PTC. For example, phosphatidylcholine-specific RHOA has been identified as indispensable in the thyrotropin-induced activation of phospholipase D in thyroid cells [22]. Src inhibitors have been proposed to be very useful in suppressing the growth of PTC [23]. Src-mediated inhibition in advanced PTC with BRAF and RAS mutations holds great promise for improving the clinical outcomes [24]. In this topic, we provided a potential novel function that is different from the traditional biological function. These findings also pave the way for future clinical applications. Functional enrichment analyses suggest that, in PTC, genes with prognosis-related AS events are associated with several energy metabolism-related pathways and therefore may provide a selective advantage to cancer cells through reprogramming of the energy metabolism. Tumor cells fuel wild growth that requires adjustments of energy metabolism [25]. The thyroid gland plays an irreplaceable role in body metabolism, owing to the various hormones produced by the thyroid. Multiple studies have reported that serine/glycine, glutamine metabolism-related protein expression is different according to the thyroid cancer subtype [26,27]. Quite surprisingly, precision metabolism target therapies of thyroid cancer have been investigated in recent decades [28–30]. Our findings proposed a panel of AS events that exert their biological functions in metabolic alterations of PTC.

The highlight of the current study was that we proposed prognostic signatures based on AS events for monitoring the prognosis of PTC patients. Owing to the favorable overall survival of PTC patients, PFI was selected as the endpoint. Recently, some multi-molecule-based signatures in PTC have been proposed. Bisarro Dos Reis M et al. generated a prognostic scoring system in well-differentiated thyroid carcinoma 21 DNA methylation probes [31]. Cai WY et al. also developed a prognostic signature by combining two mRNAs and two long noncoding RNAs for predicting the progression and prognosis of PTC [20]. Among patients with PTC, there remains a huge demand for additional tools to improve clinical management. Furthermore, different molecular biomarker-based signatures could provide different perspectives. Here, we used the LASSO Cox regression model to screen out a set of AS events to facilitate clinical transfer. Prognostic signatures we proposed displayed ideal performance in predicting the clinical outcome of patients. However, another independent cohort should be used to validate the efficiency.

An enormous number of AS events in organism cells are orchestrated by limited SFs [32]. The alteration spectrum of AS events that occur in multiple tumor types highlights splicing factors as an important mechanism of splicing deregulation in cancer [33]. Alterations of SFs in PTC are increasingly considered as independent molecules involved in carcinogenesis and progression via various mechanisms [34–36]. Splicing correlation network analysis has also clearly revealed larger regulated nodes, suggesting the prominent positions they hold in the SF-AS network. NOVA1 was highly connected in the network could contribute to the prognosis induced by splicing events. NOVA1 has been reported to play a significant role in carcinogenesis [37–39]. However, the role of NOVA1 in PTC has not been explored. Nevertheless, our algorithm suggested deregulated AS events as a hallmark of PTC. However, several limitations inevitably influenced the results' reliability. First, PTC are characterized by an indolent disease course. As a consequence, a significant proportion of patients considered to be disease-free could develop a disease recurrence in the subsequent follow-up. Second, another independent validation cohort should be used to validate in the future. Third, more in vivo and in vitro functional experiments are clearly needed to further explore the impact of dysregulated AS events and SFs in cancer development.

In summary, the current study established a detailed phenomenon base of prognosis-associated AS events in PTC patients, which is valuable in deciphering the functional contribution of AS events in PTC. These findings should facilitate the ongoing effort in developing novel genomic models for clinical cancer management. Moreover, the further identification of prognostic splicing factors and construction of the SF-AS network will pave the way for further exploration of splicing-related mechanisms.

## MATERIALS AND METHODS

### Data acquisition

TCGA SpliceSeq [40] is a data portal that provides AS profiles across 33 tumors based on TCGA RNA-seq data. SpliceSeq evaluates seven types of splice events, including AA, AD, AP, AT, ES, ME, and RI. To quantify AS events, TCGA SpliceSeq processed the percent spliced in (PSI) value for cancer research analysis, which indicates the inclusion of a transcript element divided by the total number of reads for that AS event. Alterations in PSI values range from 0 to 100 (%), which suggests a shift in splicing events. AS events with a PSI value of more than 75% in thyroid carcinoma cohort samples were downloaded from the TCGA SpliceSeq database. The AS events with standard diversion < 1 were removed.

Clinical information of PTC patients was also downloaded and extracted from the pan-cancer atlas database of TCGA [41]. The PFI was used as the endpoint for survival analysis owing to the limited number of events for overall survival. More importantly, PFI records more endpoint events during the follow-up period. Only pathologically confirmed PTC patients with both follow-up and AS event data were included for our analysis. The same TCGA ID was used to integrate clinical information and AS events data.

### Survival analysis

In the survival analysis, the follow-up periods ranged from 91 days to 5423 days after removing patients with PFIs of less than 90 days. Univariate Cox analysis was conducted to assess the relationships between the PSI value (from 0 to 100) of each AS event and the PFI of PTC patients (P < 0.05). LASSO method is a popular method for the regression of high-dimensional predictors [42]. LASSO has been extended for use in Cox regression survival analysis and is ideal for high-dimensional data. We selected the LASSO Cox regression model to determine the ideal coefficient for each prognostic feature and to estimate the deviance likelihood via 1-standard error (SE) criteria. The coefficients and partial likelihood deviance were calculated with the “glmnet” package in R.

### Functional annotation

Functional annotation for the genes with survival-associated AS events was performed by the bioinformatics tool “clusterProfiler” to comprehensively explore the dysregulated functional relevance of these genes [43]. Gene ontology (GO) and the Kyoto Encyclopedia of Genes and Genomes (KEGG) were used to assess the functional categories. GO and KEGG terms with a P-value and q-value both smaller than 0.05 were considered significant categories.

### Prognostic signature construction

The top 20 most significant AS events in univariate Cox analysis were submitted to LASSO regression Cox analysis to develop prognostic signatures based on seven types of AS events. Finally, prognostic signatures for PFI prediction were calculated by multiplying the PSI values of prognostic indictors and the coefficient assigned by LASSO Cox analysis. The evaluation of the splicing-based prognostic signature as an independent predictor was conducted by integrating the following clinical parameters into the multivariable Cox regression analysis: age (≥50 and <50), gender (male or female), AJCC TNM stage (stage III/IV or stage I/II), tumor stage (T3-T4 or T1-T2), lymph node metastasis (positive or negative), distant metastasis (positive or negative), neoplasm focus type (multifocal or unifocal) and tumor status (with tumor or tumor-free).

### SF-AS regulatory network

A compendium of 404 splicing factors was obtained from a previous study [44]. The expression profiles of SF genes were curated from the TCGA dataset. The count value of SF level-3 mRNA data was downloaded and converted to log2(count+1) for further univariate Cox analysis. We selected axes between the expression value of SFs and PSI values of prognosis-related AS events to construct the SF-AS regulatory network according to the following conditions: P value less than 0.05 and Pearson correlation coefficient more than 0.3. Then, we built the correlation plots via Cytoscape version 3.6.1.
